# Identification of Specific Cell Surface Markers on Immune Cells of Squirrel Monkeys (*Saimiri sciureus*)

**DOI:** 10.1155/2024/8215195

**Published:** 2024-03-25

**Authors:** Bharti P. Nehete, Ashley DeLise, Pramod N. Nehete

**Affiliations:** ^1^Department of Comparative Medicine, The University of Texas MD Anderson Cancer Center, Bastrop, Texas, USA; ^2^The University of Texas Graduate School of Biomedical Sciences, Houston, Texas, USA

## Abstract

Nonhuman primates are an important experimental model for the development of targeted biological therapeutics because of their immunological closeness to humans. However, there are very few antibody reagents relevant for delineating the different immune cell subsets based on nonhuman primate antigens directly or with cross-reactivity to those in humans. Here, we report specific expression of HLA-DR, PD-1, and CD123 on different circulating immune cell subsets in the peripheral blood that included T cells (CD3+), T cells subsets (CD4+ and CD8+), B cells (CD20+), natural killer (NK) cells (CD3–CD16+), and natural killer T cells (CD3+CD16+) along with different monocyte subsets in squirrel monkey (*Saimiri sciureus*). We established cross-reactivity of commercial mouse antihuman monoclonal antibodies (mAbs), with these various immune cell surface markers. These findings should aid further future comprehensive understanding of the immune parameters and identification of new biomarkers to significantly improve SQM as a model for biomedical studies.

## 1. Introduction

The National Center for Research Resources of the National Institutes of Health (NIH) recognized the importance of the squirrel monkey in turn establishing a self-sustaining breeding colony to support the needs of NIH grantees in using squirrel monkeys for a multidisciplinary research program that will aid in better understanding the biology and behavior of squirrel monkeys (1980–1985) [1]. The squirrel monkey (genus *Saimiri*) belonging to the family Cebidae, is a neotropical primate native to Central and South America. Squirrel monkeys are commonly used as a model in biomedical research since they have remarkably similar immune systems to humans [2]. Like humans, aging monkeys show physical losses in activities [3–6]. Many biomedical studies employing Squirrel monkeys focus on infectious disease, prion infectivity, gene expression, cancer treatments, reproductive physiology, viruses, and role in parasitic diseases [2, 6–13]. Species differences regarding disease susceptibility has been overlooked until recently [8, 14]. Adult and neonate squirrel monkeys may be a suitable model for testing HBV therapeutics, compared to Woolly Monkey HBV (WMHBV) that exhibit prolonged viremia lasting 6–8 months which is twice the duration of viremia achieved in other nonhuman primates [15]. Experimental Zika virus infection of neotropical primates, both squirrel and owl monkeys were observed in the absence of detectable disease, but seroconversion occurred by day 28 [16]. Squirrel monkeys are also an important model in the development of pharmacological and physiotherapeutic interventions to improve motor recovery after stroke as described by Nudo et al. [17]. The squirrel monkey model can be useful for studying Cerebral Amyloid Angiopathy (CAA), pathogenesis, and long-term effect of Amyloid Related Imaging Abnormalities (ARIA) for testing the safety and efficacy of emerging therapeutic for Alzheimer's disease [18]. Similarly, we also observed age-related Alzheimer's disease-related pathologies in squirrel monkey showing improvement by stimulating innate immunity via CpG administration [19]. Comparably, squirrel monkeys are a natural host of *Herpesvirus saimiri* (saimiri herpesvirus 2) which can be isolated from blood samples obtained from healthy squirrel monkeys [20, 21].

Squirrel monkeys (*Saimiri* spp.) are the most commonly used neotropical primates in biomedical research in the United States. Their physical characteristics, including small size and ease of handling, contribute to their desirability as research subjects. The mean body weight of adult squirrel monkeys is less than 1 kg compared to female rhesus monkeys, which usually weigh 4–5 kg. As a result, much smaller doses of synthesized compounds are necessary when using squirrel monkeys to evaluate new drugs, which is an important advantage when studies require the administration of expensive compounds. Squirrel monkeys easily adapt to laboratory housing as well as can be maintained in smaller and less expensive spaces as opposed to larger primates such as macaques and baboons. This characteristic is especially important for facilities with space limitations. The substantial number of studies using the squirrel monkey over the past 40 years has provided a wealth of basic information about the biology of this neotropical primate. This information provides important baseline data for studies being conducted today and for future studies. In addition, squirrel monkeys are small and can be easily managed in comparison to large Old-World primates such as the rhesus macaque and chimpanzee. Although research methods and approaches have changed radically over the past decade with advances in molecular and cellular biology, the use of squirrel monkeys has remained constant. However, the lack of specific immunological reagents for neotropical monkeys, such as *Saimiri sciureus*, is still a major factor limiting studies in these models. The present investigation is intended to circumvent this obstacle by selecting immunological reagents directed toward homologous human markers, with strong cross-reactivity.

## 2. Study Population

### 2.1. Animals, Care, Diet, and Housing

Squirrel monkeys were socially housed at the University of Texas, MD Anderson Cancer Center at Keeling Center, Bastrop, TX, used in this study. Animals were maintained as described previously [22].

Animals had *ad libitum* access to the new world Primate Diet (Purina #5049) and water. In addition, they were fed either a fresh fruit or vegetable daily. Specialty foods, such as seeds, peanuts, raisins, yogurt, cereals, frozen juice cups, and peanut butter, were distributed daily as enrichment. The animals were never deprived of food or water. Animals were also given destructible enrichment manipulanda and various travel/perching materials on a rotating basis to promote typical species behavior.

## 3. Methods

### 3.1. Blood Collection

Blood samples (2–3 mL) were collected in EDTA-coated collection tubes from the femoral veins of study animals in the morning (8–9 AM) before the animals were fed. The monkeys were examined by veterinarians during the study period and determined to be healthy.

### 3.2. Antibodies, Reagents, and Flow Cytometry

A series of commercially available human monoclonal antibodies that cross-react with NHP mononuclear cells were used in flow cytometry analyses, as described previously [22]. A cocktail of monoclonal antibodies against CD3 (clone SP-34), CD4 (clone L200), CD8 (clone 3B5), CD20 (clone L27), CD14 (clone M5E2), CD16 (clone 3G8), CD123 (clone 7G3), PD-1 (clone EH12.1), and HLA-DR (clone L243) with appropriate isotype-control antibodies were used (Table 1). One hundred microliters of EDTA blood was incubated with the cocktail of antibodies for 15 min at room temperature in the dark. Red blood cells were then lysed with a 1 × RBC lysing solution (Becton Dickinson, USA) following the manufacturer's instructions. Immediately following, the samples were washed thoroughly in FACS buffer (1 × phosphate-buffer saline (PBS), FBS, and sodium azide) by centrifugation; then cell pellets were suspended in 1% paraformaldehyde buffer (300 *µ*L) and acquired on a Celesta flow cytometer (BD Biosciences, San Jose, CA, USA). All samples acquired in this study were compensated using the single-color stained beads. Lymphocytes, monocytes, NK, and natural killer T (NKT) cells were gated on a forward scatter versus side scatter plot, as shown in Figures S1–S5. FlowJo software (Tree Star, Inc., Ashland, OR USA) was used to analyze HLA-DR, PD-1, and CD123 expression on lymphocytes, monocytes, NK, and NKT cell subsets.

### 3.3. PBMC Preparation

Peripheral blood mononuclear cells (PBMCs) were isolated by standard Ficoll–Hypaque 1640 density gradient (Sigma–Aldrich, USA) centrifugation and subsequently used for *in vitro* analyses including viability and functional assays.

### 3.4. In Vitro Mitogen Stimulation

Aliquots of PBMC (1 × 10^5^) were stimulated with Concanavalin A (Con A, Sigma–Aldrich, USA) (2 *µ*g/mL) for 72 hr in the presence and absence of anti-PD-1 (5 *µ*g/mL) blocking antibody. An Isotype control antibody (IgG1), at same concentration of anti-PD-1, was used as a control. Stimulation index was calculated by dividing the absorbance (A540) values for PBMC treated with Con A (+Con A) with those without treatment (−Con A). Similar analyses were performed for PBMC (1 × 10^5^) stimulated with Con A (2 *µ*g/mL) for 72 hr in presence and absence of anti-HLA-DR antibody (Clone L243; 5 *µ*g/mL) or an isotype control antibody (IgG2a).

### 3.5. ELISPOT Assay for Detecting Mitogen-Specific IFN-*γ* Producing Cells

Freshly isolated, PBMC (50,000) were stimulated with Con A (2 *µ*g/mL) for 40 hr in the presence and absence of HLA-DR (clone L243) blocking antibody or an Isotype control antibody (IgG2a), each at 5 *µ*g/mL in a precoated IFN-*γ* ELISPOT plate using the methodology reported earlier [22]. The ELISPOT plates were developed and read on an IRIS ELISPOT reader (Mabtech IRIS, Cincinnati, USA).

### 3.6. Statistical Analysis

Data were evaluated using GraphPad Prism 9 software. Ordinary one-way ANOVA with a Brown–Forsythe test was used to determine statistical significance with *p* values < 0.05 considered statistically significant.

## 4. Results

**Figure 1:**
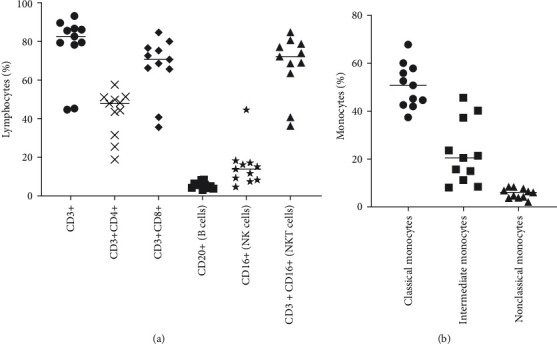
Enumeration of circulating levels of lymphocyte subsets. The different lymphocyte subsets (a) and monocyte subsets (b) as shown are identified by flow cytometry, and the frequencies are shown.

We determined the levels of major lymphocyte subsets in the peripheral blood of squirrel monkeys using flow cytometry to profile different lymphocyte subsets that included total T cells (CD3+), helper T cells (CD3+CD4+), cytotoxic T cells (CD3+CD8+), B cells (CD20+), natural killer (NK) cells (CD3–CD16+), and NKT cells (CD3+CD16+) (Figure 1(a)). Additionally, we identified different monocyte populations as classical (CD14^++^CD16^−^), intermediate (CD14^++^CD16^+^), and nonclassical (CD14^+^CD16^+^) subsets (Figure 1(b)).

It has been reported in mouse models of Alzheimer's disease by Schwartz et al. [23] that immune checkpoint blockade targeting the programed death-1 (PD-1) pathway improved the immune responses that lead to disease modification. Since our earlier studies showed that innate immunity stimulation via treatment with CpG oligodeoxynucleotides ameliorates Alzheimer's disease pathology in aged squirrel monkeys [19], we tested the modulation of the expression of PD-1 and HLA-DR on immune cells of squirrel monkey as a potential underlying mechanism.

**Figure 2:**
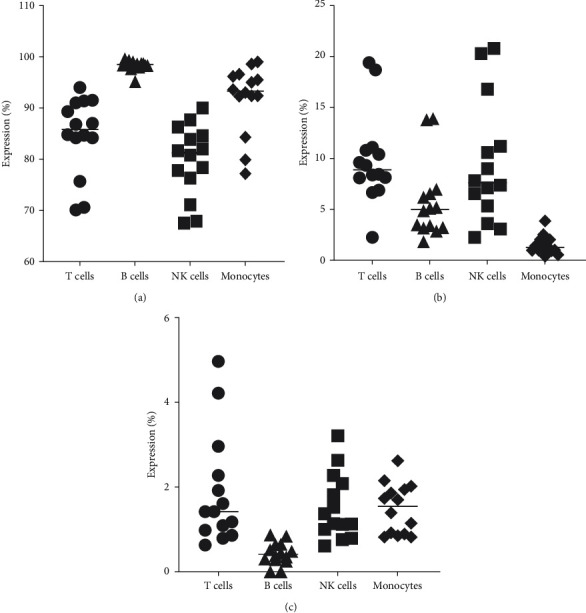
Expression on HLA-DR+ (a), PD-1+ (b), and CD123+ (c) on T cells, B cells, NK cells, and monocytes of squirrel monkey.

For this, we first determined the expression of HLA-DR+ (Figure 2(a)), PD-1+ (Figure 2(b)), and CD123+ (Figure 2(c)) on different squirrel monkey immune cells, using flow cytometry.

**Figure 3:**
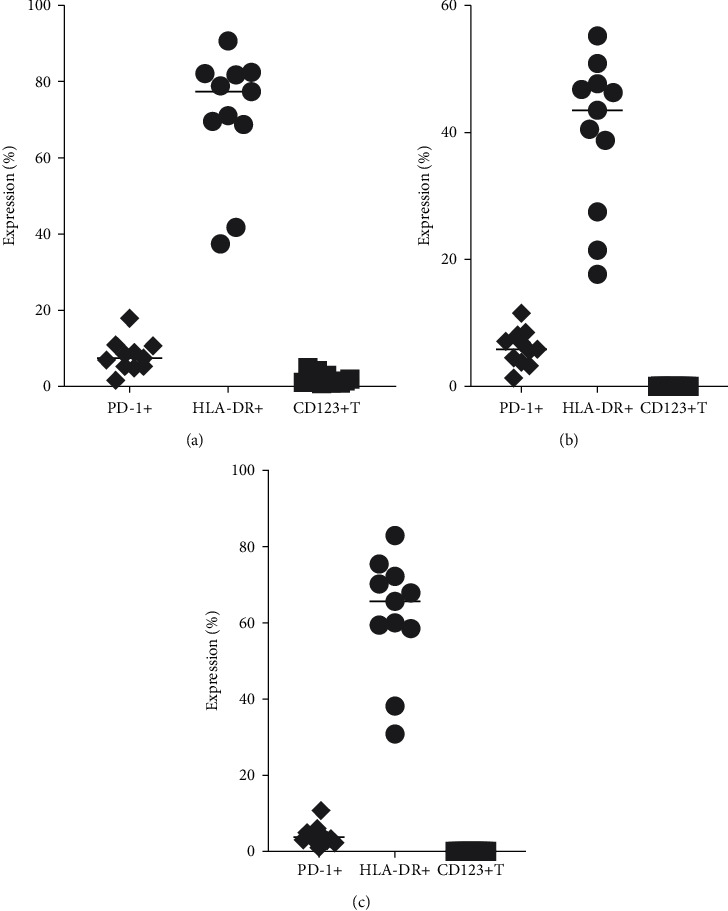
Expression of PD-1+, HLA-DR+, and CD123+ on subsets of T cells: CD3+ T cells (a), CD4+ T cells (b), and CD8+ T cells (c).

Additionally, the next step was to show expression of HLA-DR+, PD-1+, and CD123+ on subsets of specific T cells: CD3+ (Figure 3(a)), CD4+(Figure 3(b)), and CD8+ (Figure 3(c)).

**Figure 4:**
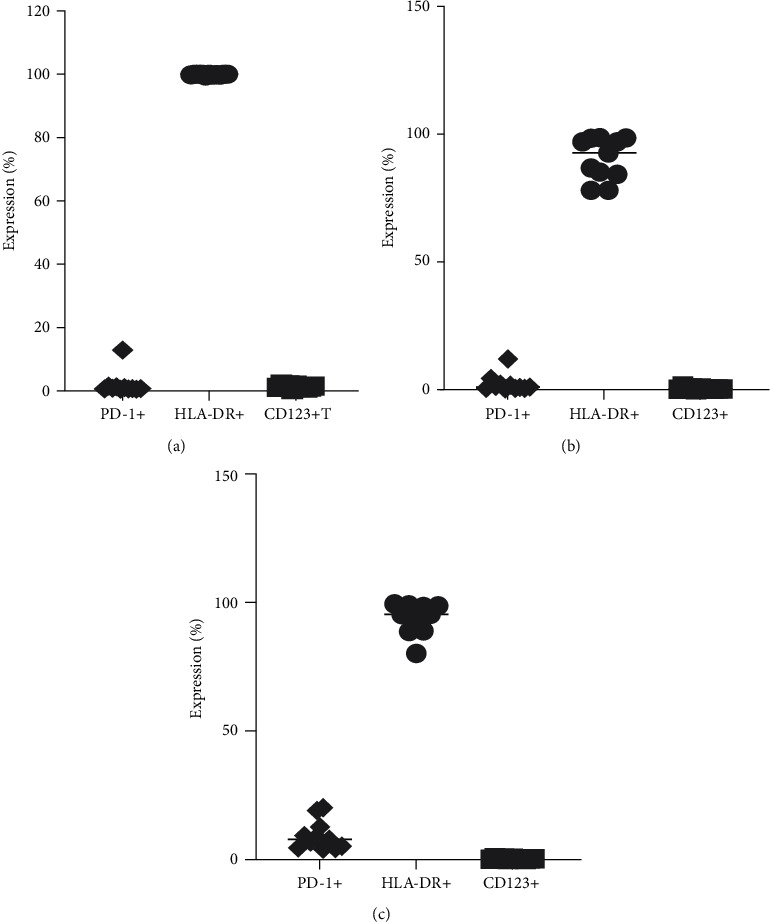
Expression of PD-1, HLA-DR, and CD123 on B cells: CD20+ B cells (a), NK cells (b), and NKT cells (c).

We also analyzed the expression of HLA-DR+, PD-1+, and CD123+ on B cells, NK cells, and NKT cells by incorporating all cell surface markers simultaneously. We define B cells (CD20+) (Figure 4(a)), NK (CD16+) (Figure 4(b)), and NKT (CD13+CD16+) (Figure 4(c)). The specificity of staining for the various markers was found according to the isotype control antibody used for each pair of combination markers. A scheme of the expression of HLA-DR+, PD-1+, and CD123+ on B, NK, and NKT cells along with compensation using single-color stained cells in whole blood of squirrel monkey by flow cytometry is shown in Figure 4.

**Figure 5:**
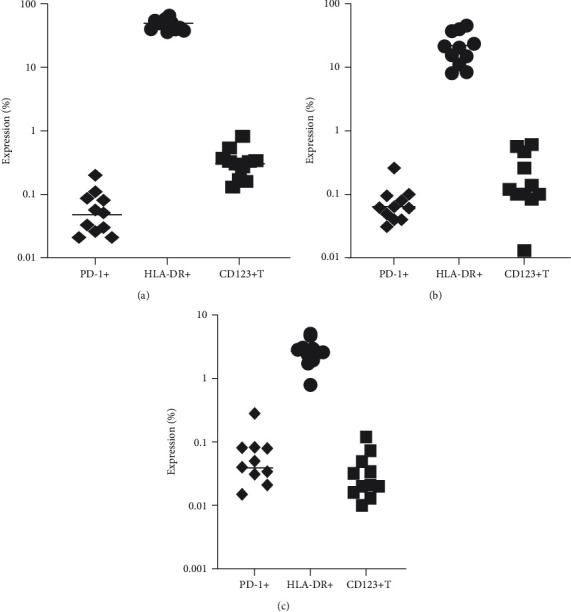
Expression of PD-1, HLA-DR, and CD123 on classical monocytes (a) CD14 and CD16, intermediate (b), and nonclassical monocytes (c).

The logical gating was used to identify the expression of HLA-DR, PD-1, and CD123 on the monocyte CD14+ (Figure 5) populations in whole blood. Using FlowJo analysis, we found that although classical monocytes (Figure 5(a)) are defined with high purity using CD14 and CD16, intermediate (Figure 5(b)) and nonclassical monocytes (Figure 5(c)) defined using CD14 and CD16 alone are frequently contaminated, with average intermediate and nonclassical monocyte purity of ∼86.0% and ∼87.2%, respectively.

### 4.1. Effect of Activation on the Expression Levels of HLA-DR, PD-1, and CD123 on Different Immune Cells Subsets

**Figure 6:**
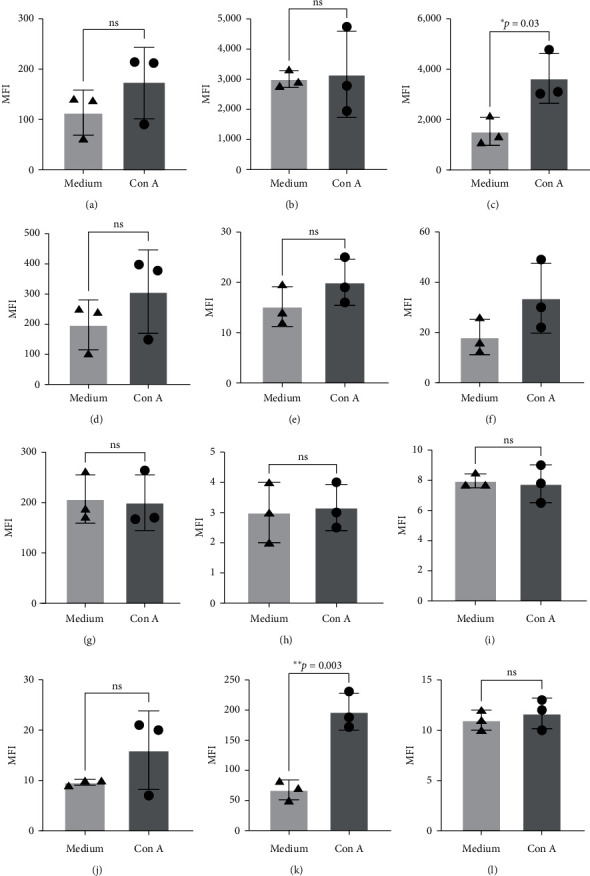
Mean fluorescence intensity (MFI) of HLA-DR on T cells (a), HLA-DR on B cells (b), HLA-DR on monocytes (c), HLA-DR on NK cells (d); PD-1 on T cells (e), PD-1 on B cells (f), PD-1 on monocytes (g), PD-1 on NK cells (h); and CD123 on T cells (i), CD123 on B cells (j), CD123 on monocytes (k), CD123 on NK cells (l). Values are reported as not significant (ns),  ^*∗*^*p*=0.032, or  ^*∗∗*^*p*=0.0032.

Results from animal studies suggest that PD-1/PD-L1 suppresses memory T cell responses, including proliferation and cytokine production [24]. Blocking the PD-1/PD-L1 pathway potentially results in an increase in T cell activation [25]. To begin to understand this phenomenon in the squirrel monkeys, we first determined the expression levels of PD-1 along with CD123 and HLA-DR on the different immune cell subsets after activation with Con A in the absence and presence of blocking antibodies specific to each of these markers. After treatment of PBMC with Con A for 24 hr, we observed no significant changes in the levels of any of the three markers on T, B, and NK cells (Figures 6(a), 6(b), and 6(d)). However, we observed significant increase in the expression of HLA-DR (Figure 6(c)) and CD123 on monocytes (Figure 6(k)).

### 4.2. Effect of Immune Cell Functions after Specific Binding of Antibodies

**Figure 7:**
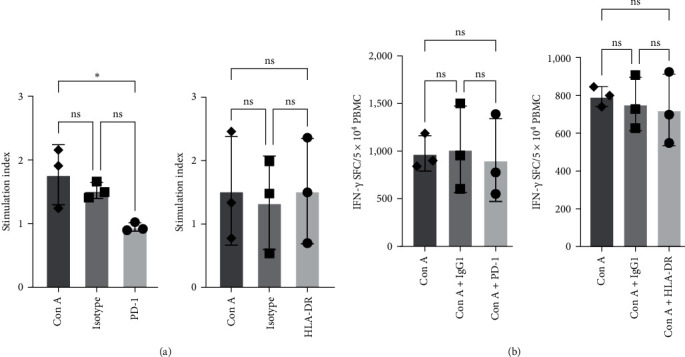
Blocking of steady state PD-1 and HLA-DR expression on mitogen stimulation of squirrel monkey PBMC (a) and IFN-*γ* ELISPOT (b): PBMC (1 × 10^5^) were stimulated with Con A (2 *µ*g/mL) for 72 hr in the presence and absence of PD-1 (5 *µ*g/mL) or HLA-DR (Clone L243; 5 *µ*g/mL) blocking antibodies. Stimulation index was calculated by division of absorbance (A540) of PBMC (+Con A) with absorbance of PBMC (−Con A) (a). PBMC (50,000) were stimulated with Con A (2 *µ*g/mL) for 40 hr in the presence and absence of PD-1 (1 *µ*g/mL) or HLA-DR (clone L243; 5 *µ*g/mL) blocking antibodies for IFN-*γ* ELISPOT (b). In both experiments, an isotype control (IgG1 or IgG2a for PD-1 and HLA-DR, respectively) at the same concentration of PD-1 or HLA-DR was used as a control. Results from three animals are shown. Values are reported as not significant (ns),  ^*∗*^*p*=0.032, or  ^*∗∗*^*p*=0.0032.

We evaluated for changes in the functionality of immune cells, in terms of proliferation and cytokine production, by stimulating the cells with Con A in the presence and absence of antibodies specific to PD-1, HLA-DR, and CD123 (Figure 7). Aliquots of PBMC were stimulated with Con A (2 *µ*g/mL) for 72 hr with blocking antibodies or isotype control (IgG1) antibodies. We observed no significant blocking of proliferation in the presence of anti-HLA-DR antibody, but we saw significant reduction with anti-PD-1 antibody (Figure 7(a)).

We also assessed cytokine production as a measure of activation using the IFN-*γ* ELISPOT assay. For this, aliquots of PBMC (50,000) were stimulated with Con A (2 *µ*g/mL) for 40 hr in the presence and absence of antibodies specific to PD-1 (1 *µ*g/mL) and HLA-DR (clone L243; 5 *µ*g/mL) along with an isotype control (IgG1) antibody. We observed no significant inhibition in IFN-*γ* production in the ELISPOT assay (Figure 7(b)).

## 5. Discussion

The squirrel monkey (SQM) serves as an important nonhuman primate model for drug development, specifically in the next generation of targeted therapeutics that affect specific pathways and cell types rather than a broad activation of the immune system. Toward this goal, we have been diligently conducting studies to create a comprehensive atlas of antibodies that can be used for phenotypic and functional characterization of the immune cells in squirrel monkey. We reported earlier cross-reactivity between several commercially available mouse antihuman monoclonal antibodies (mAbs) conjugated to fluorochromes and peripheral blood major leukocyte surface antigens in NHP using whole blood flow cytometric analysis [26]. The present study is focused on determining the expression of HLA-DR, PD-1, and CD123 on SQM monocytes, NK, and NKT cells in addition to B cells and T cell subsets.

In squirrel monkeys, as in humans, monocytes are a major component of peripheral blood, accounting for ≈10% of all circulating leukocytes. They are divided into three major populations by flow cytometry using a quadrant-based gating scheme with CD14 and CD16 to distinguish among the three monocyte subsets defined as classical (CD14^++^CD16^−^), intermediate (CD14^++^CD16^+^), and nonclassical (CD14^+^CD16^+^) constituted at 68.3%, 5.7%, and 20.2% of total blood monocytes, respectively [27, 28]. Each of these subsets is distinguished from each other by the expression of distinct surface markers as well as their functions in homeostasis and disease. Intermediate monocytes are more abundant in bacterial sepsis [29], dengue fever [30], Crohn's disease [31], cardiovascular disease (CAD) [32], and rheumatoid arthritis [33], whereas the nonclassical monocytes are more prevalent in periodontitis [34] yet reduced in stroke [35]. The contribution of human monocytes to the progression of these diseases highlights their candidacy as potential therapeutic cell targets [36]. Alterations in monocyte subset frequencies are associated with clinical outcomes, including cardiovascular disease, in which circulating intermediate monocytes independently predict cardiovascular events. However, delineating mechanisms of monocyte function is hampered by inconsistent results among studies.

Likewise, we observed NK cells, a type of cytotoxic lymphocyte in squirrel monkey representing 5%–20% of all circulating lymphocytes, similar to that in humans [37]. The role of NK cells is analogous to that of cytotoxic T cells in the vertebrate adaptive immune response. NK cells are unique as they can recognize and kill stressed cells in the absence of MHC restriction, allowing for a much faster immune reaction. They were named “natural killers” because of the notion that they do not require activation to kill cells that are missing “self” markers of MHC class 1 [38]. This role is especially important because harmful cells that may be missing MHC I markers cannot be detected or destroyed by other immune cells, such as T lymphocyte cells.

In addition, NKT cells are a subset of CD1d-restricted T cells at the interface between the innate and adaptive immune system. NKT cells are an extremely rare subset of T cells, typically less than 1% in peripheral blood of humans and nonhuman primates. NKT cells are rapid responders of the innate immune system and mediate potent immunoregulatory and effector functions in a variety of disease settings [39]. For example, NKT cells are known to develop increases in activation and effector function within the breast tumor microenvironment. Due to a lack of cross-reactivity of CD1d tetramer in SQM, as described previously, we define CD3+CD16+ cells as NKT cells.

We observed PD-1 expression on the cell surface of T cells, NK cells, NKT cells, B cells, and monocyte populations in squirrel monkey as reported earlier on human cells [40, 41]. PD-1 expression on naïve T cells is induced upon TCR activation [42]. This transient expression decreases in the absence of TCR signaling but is maintained upon chronic activation from a persisting epitope target such as in chronic viral infections or in cancer [43]. PD-1 ligation to its ligands PD-L1 and PD-L2 impairs TCR signaling and CD28 costimulation [44, 45].

As in humans, we also detected HLA-DR expression on effector T cell, B cell, and NK cells in squirrel monkey. In humans, HLA-DR expression upon their activation has been intensively described in some diseases, such as autoimmune diseases and viral infections [46–48]. The increase of HLA-DR on the surface of cytotoxic T cells (CTLs), upon stimulation, could also be required to boost an effective immune response. HLA-DR (a human leukocyte antigen) is a class II MHC molecule, normally expressed in professional antigen presenting cells, namely, CTLs were found to be upregulated 24/48 hr after activation of these cells and is associated with increased IFN-*γ* production [49–52].

Lastly, we observed CD123 expression on squirrel monkey immune cells as observed on human B cells and NK cells in multiple hematolymphoid neoplasms, including acute myeloid leukemia, blastic plasmacytoid dendritic cell neoplasm, acute lymphoblastic leukemia, hairy cell leukemia, and malignant Hodgkin lymphoma [53, 54]. Recent studies indicate that CD123 is overexpressed in various hematologic malignancies, including acute myeloid and B-lymphoid leukemias, blastic plasmacytoid dendritic neoplasms (BPDCN), and hairy cell leukemia. Since CD123 is a membrane receptor, they are targeted for using the natural ligand or neutralizing monoclonal antibodies, showing promising antitumor activity in BPDCN and AML patients [55].

Overall, we facilitated the evaluation of the immunomodulatory activity of polyclonal activator Con A, treating squirrel monkey PBMCs and observing that increased expression of several cell surface molecules may lead to various immune responses. Our findings highlight the value of nonhuman primates and more specifically squirrel monkey, as valuable models for understanding the human immune system. Based on the association or correlation of the kinetics of activation marker expression, the result of our study defines the measurement of T cell activation and provides a comprehensive review to serve as a reference for monitoring lymphocyte function in clinical study samples. Overall, this study has revealed the cellular composition of peripheral blood in terms of lymphocyte populations using flow cytometry-based measurements of the immune systems in the squirrel monkeys.

## Figures and Tables

**Table 1:** List of human monoclonal antibodies used for analysis.

	Antibody	Color	Clone	Catalog #	Isotype	Company
1	CD3	FITC	SP34.2	556611	Mouse IgG1	BD Pharmingen
2	CD4	PerCP	L200	550631	Mouse IgG1,_k_	BD Pharmingen
3	CD8	PE	3B5	MHCD0804	Mouse IgG2a	Life Technologies
4	CD14	AF700	M5E2	557923	Mouse IgG2a,_k_	BD Pharmingen
5	CD16	BV650	3G8	563692	Mouse IgG1,_k_	BD Pharmingen
6	CD20	APC	L27	340941	Mouse IgG1	BD
7	CD123	PE-CF594	7G3	562391	Mouse IgG2a,_k_	BD Horizon
8	HLA-DR	BV605	L243	307639	Mouse IgG2a,_k_	Bio Legend
9	PD-1	BV421	EH12.1	565935	Mouse IgG1,_k_	BD Horizon

## Data Availability

The data used to support the findings of this study are included within the article.

## Supplementary Materials

Supplementary Materials Gratetegy strategy for T cell, B cell, NK cell, monocyte, and monocyte subsets.
