# Supplementary material for: Identification of Specific Cell Surface Markers on Immune Cells of Squirrel Monkeys (Saimiri sciureus)
Source: J Immunol Res. 2024 Mar 25;2024:8215195. doi: 10.1155/2024/8215195 (PMC10985276; doi:10.1155/2024/8215195)
Supplement: Supplementary Materials — Gratetegy strategy for T cell, B cell, NK cell, monocyte, and monocyte subsets. [file 8215195.f1.pptx]

## Slide 1
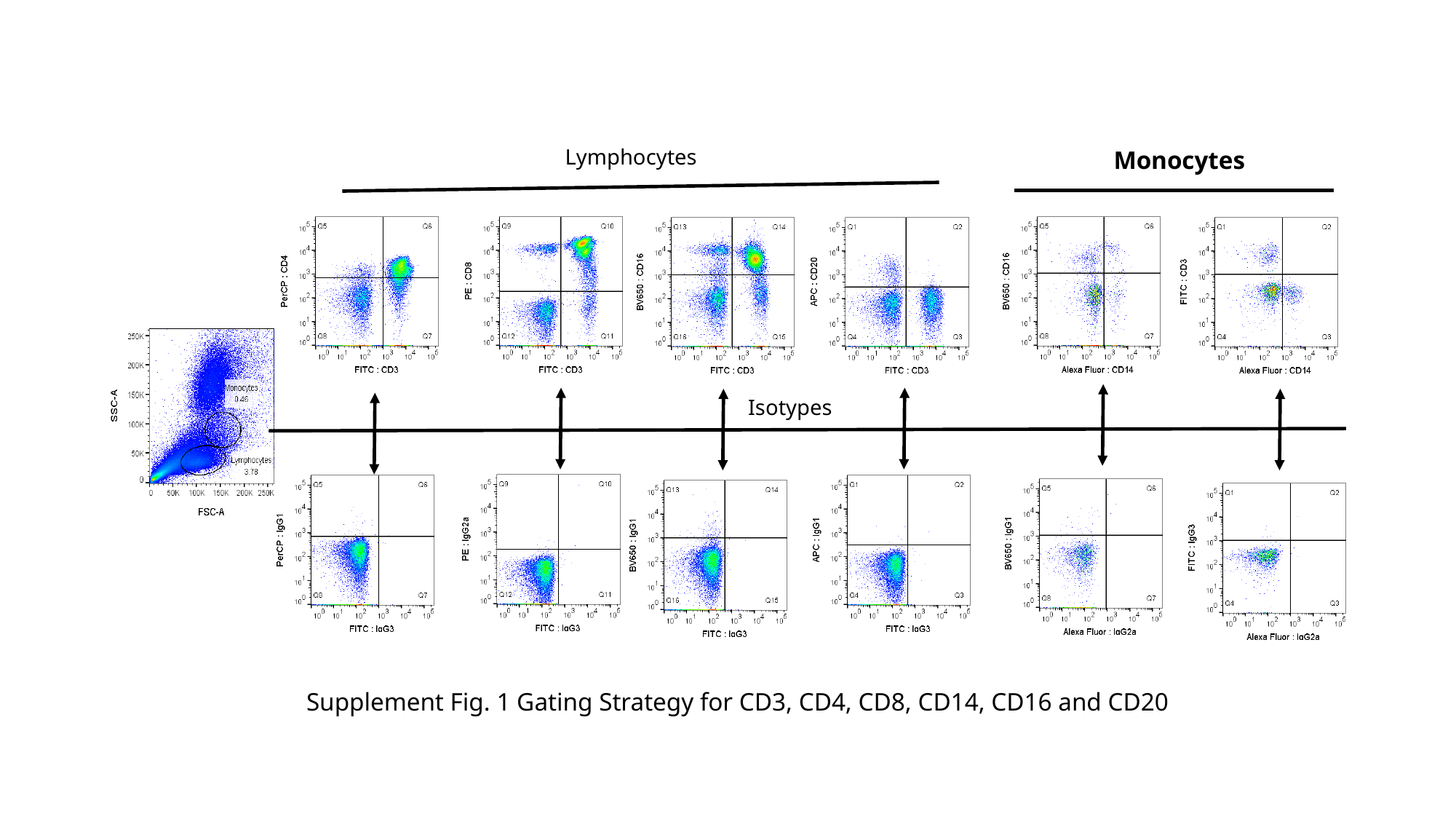

Monocytes
Lymphocytes
Isotypes
Supplement Fig. 1 Gating Strategy for CD3, CD4, CD8, CD14, CD16 and CD20

## Slide 2
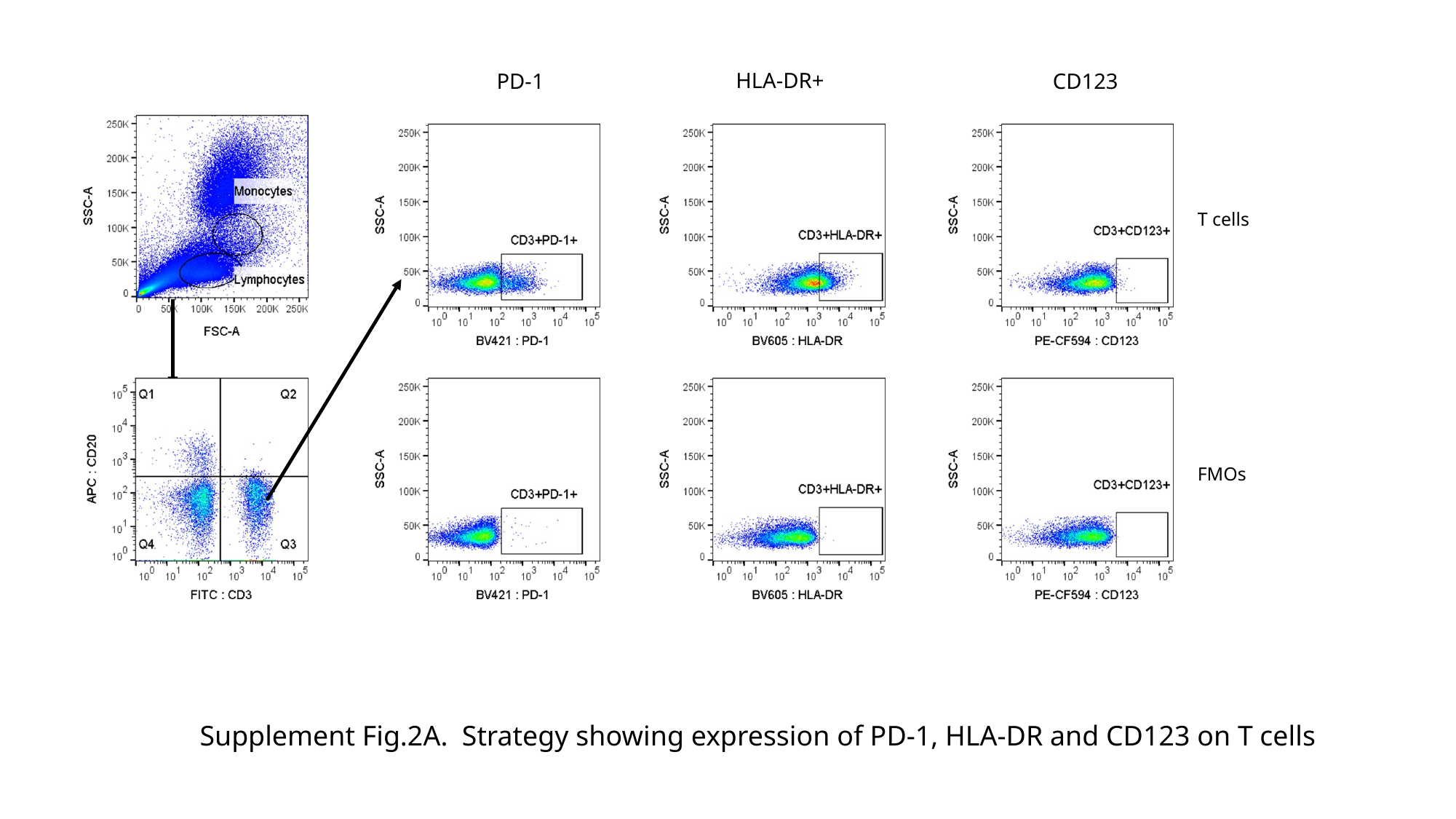

HLA-DR+
PD-1
CD123
T cells
FMOs
Supplement Fig.2A. Strategy showing expression of PD-1, HLA-DR and CD123 on T cells

## Slide 3
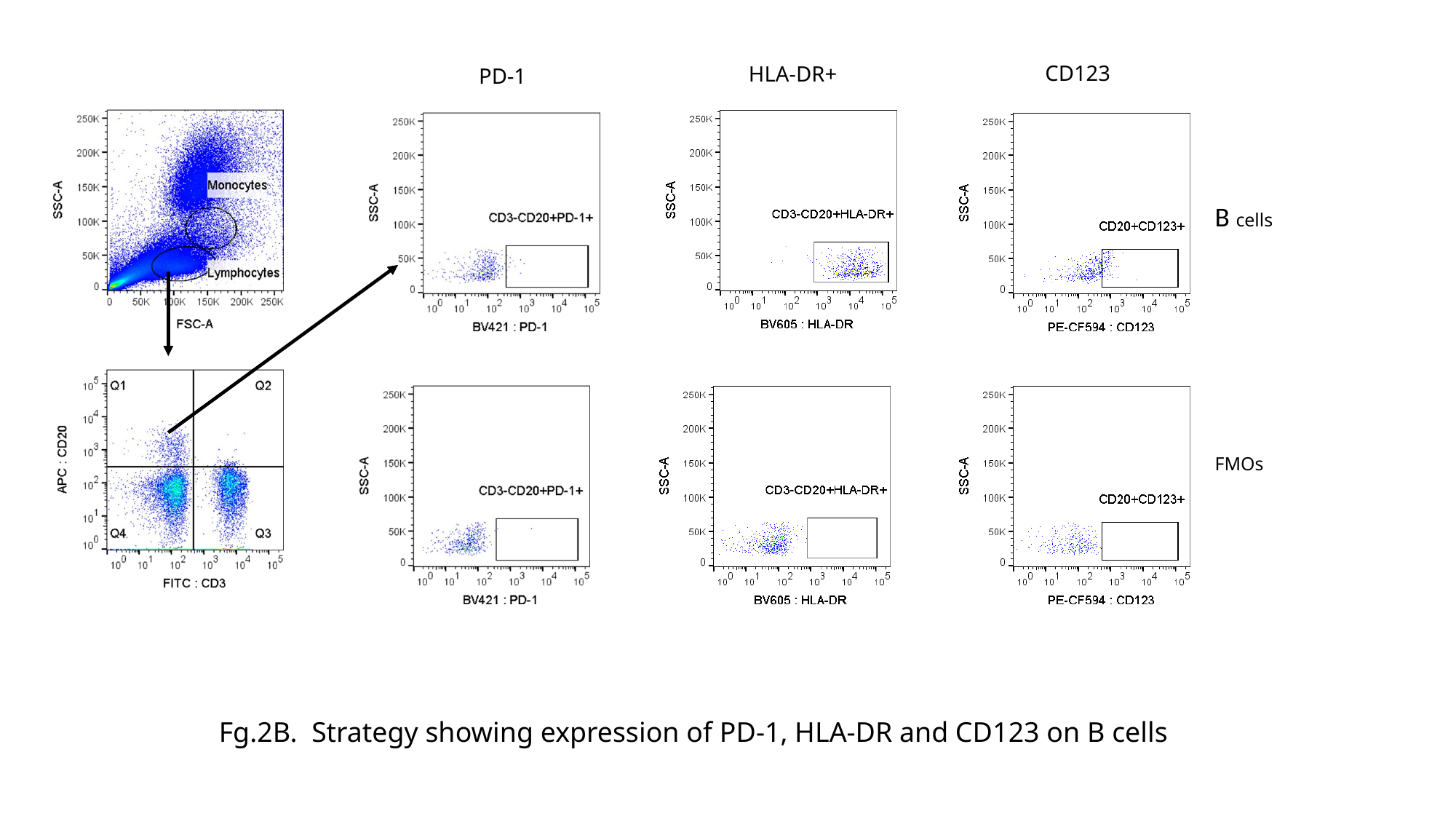

CD123
HLA-DR+
PD-1
B cells
FMOs
Fg.2B. Strategy showing expression of PD-1, HLA-DR and CD123 on B cells

## Slide 4
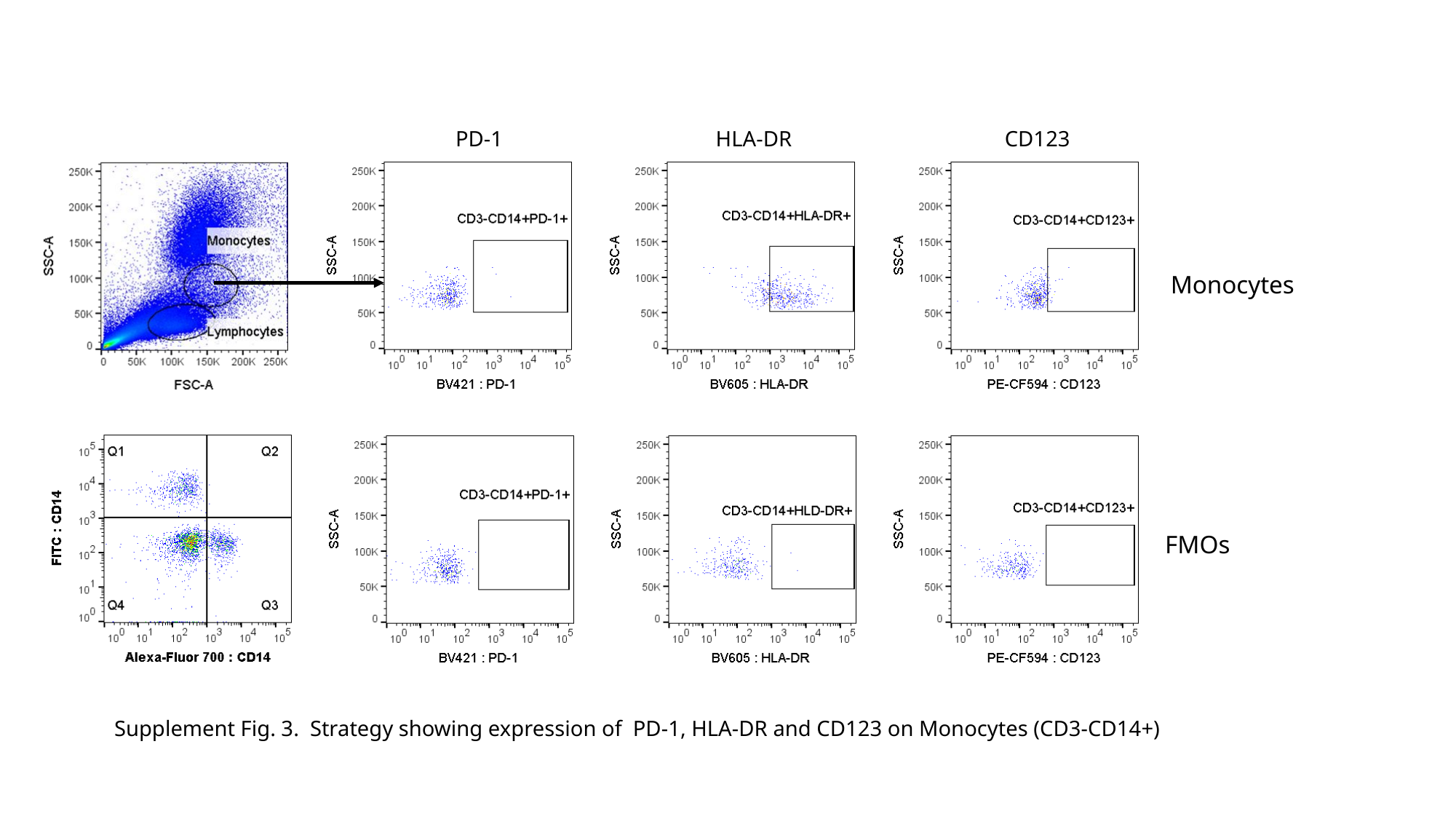

PD-1
HLA-DR
CD123
Monocytes
FMOs
Supplement Fig. 3. Strategy showing expression of PD-1, HLA-DR and CD123 on Monocytes (CD3-CD14+)

## Slide 5
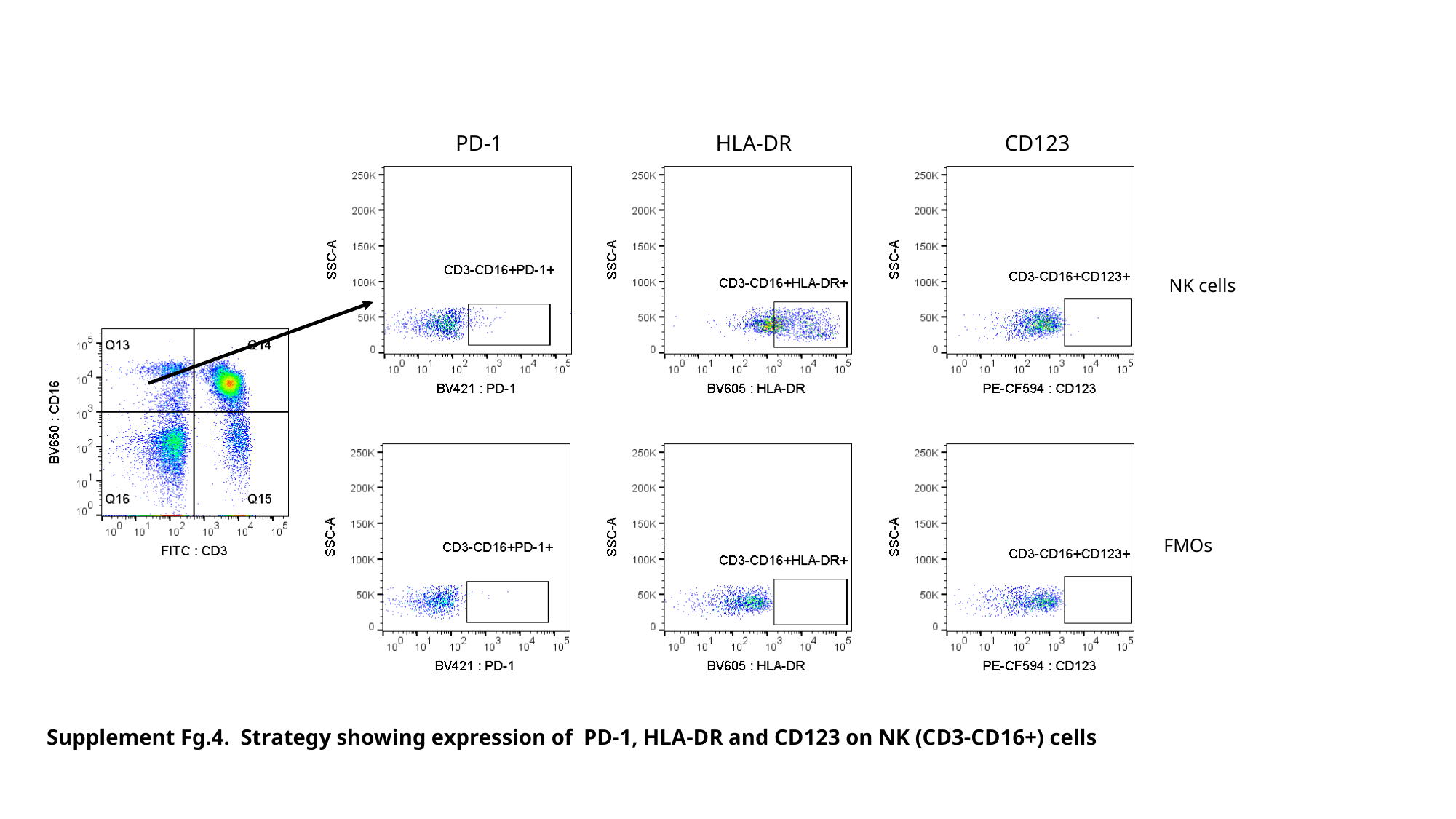

PD-1
HLA-DR
CD123
NK cells
FMOs
Supplement Fg.4. Strategy showing expression of PD-1, HLA-DR and CD123 on NK (CD3-CD16+) cells

## Slide 6
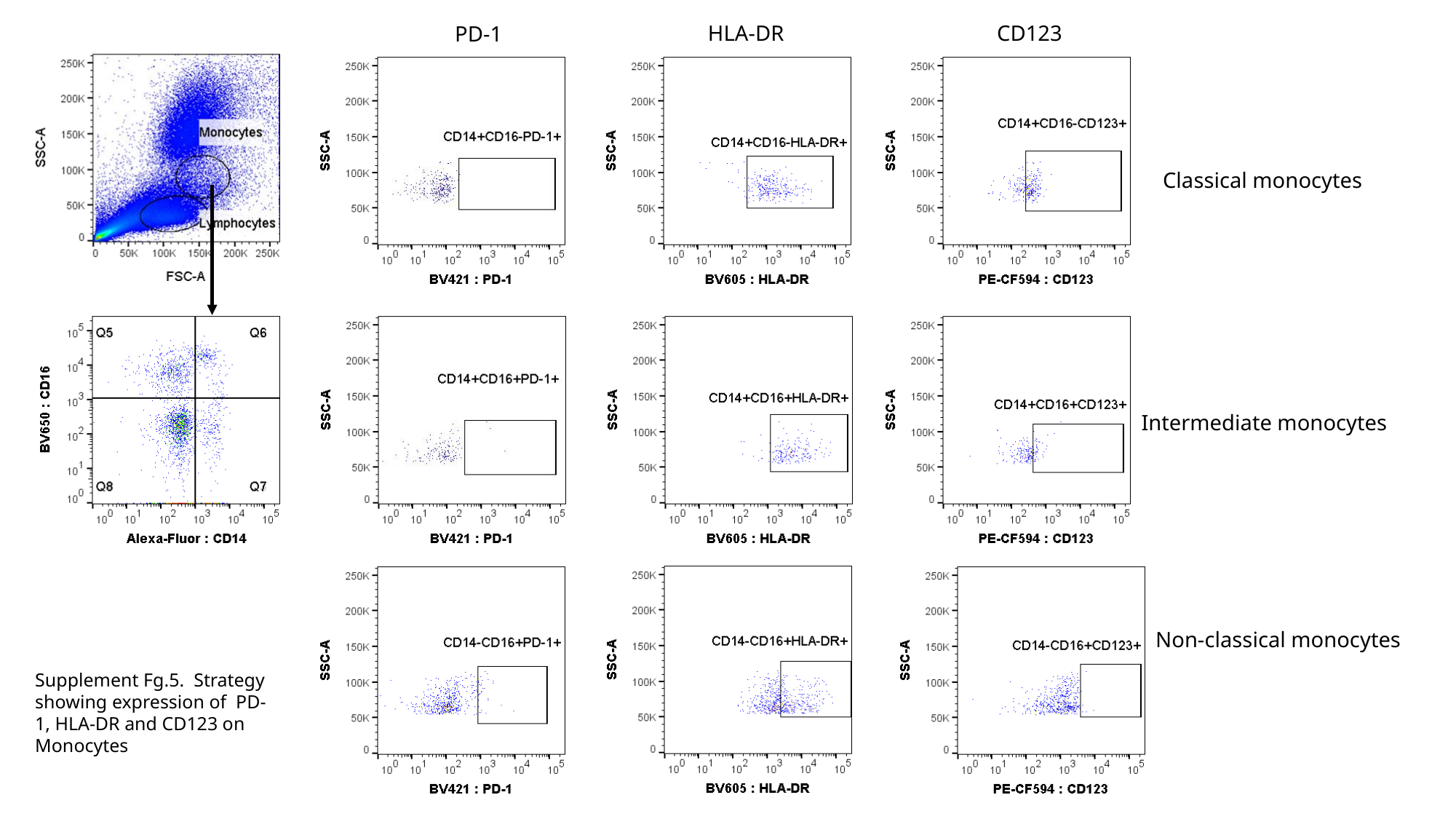

HLA-DR
CD123
Classical monocytes
Intermediate monocytes
Non-classical monocytes
Supplement Fg.5. Strategy showing expression of PD-1, HLA-DR and CD123 on Monocytes
PD-1
